# Deqformer: high-definition and scalable deep learning probe design method

**DOI:** 10.1093/bib/bbae007

**Published:** 2024-02-01

**Authors:** Yantong Cai, Jia Lv, Rui Li, Xiaowen Huang, Shi Wang, Zhenmin Bao, Qifan Zeng

**Affiliations:** MOE Key Laboratory of Marine Genetics and Breeding & Fang Zongxi Center for Marine Evo-Devo, College of Marine Life Sciences, Ocean University of China, Qingdao 266003, China; MOE Key Laboratory of Marine Genetics and Breeding & Fang Zongxi Center for Marine Evo-Devo, College of Marine Life Sciences, Ocean University of China, Qingdao 266003, China; MOE Key Laboratory of Marine Genetics and Breeding & Fang Zongxi Center for Marine Evo-Devo, College of Marine Life Sciences, Ocean University of China, Qingdao 266003, China; MOE Key Laboratory of Marine Genetics and Breeding & Fang Zongxi Center for Marine Evo-Devo, College of Marine Life Sciences, Ocean University of China, Qingdao 266003, China; MOE Key Laboratory of Marine Genetics and Breeding & Fang Zongxi Center for Marine Evo-Devo, College of Marine Life Sciences, Ocean University of China, Qingdao 266003, China; Laboratory for Marine Biology and Biotechnology, Laoshan Laboratory, Qingdao 266237, China; Southern Marine Science and Engineer Guangdong Laboratory, Guangzhou, China; Key Laboratory of Tropical Aquatic Germplasm of Hainan Province, Sanya Oceanographic Institution, Ocean University of China, Sanya 572000, China; Southern Marine Science and Engineer Guangdong Laboratory, Guangzhou, China; Key Laboratory of Tropical Aquatic Germplasm of Hainan Province, Sanya Oceanographic Institution, Ocean University of China, Sanya 572000, China; MOE Key Laboratory of Marine Genetics and Breeding & Fang Zongxi Center for Marine Evo-Devo, College of Marine Life Sciences, Ocean University of China, Qingdao 266003, China; Laboratory for Marine Biology and Biotechnology, Laoshan Laboratory, Qingdao 266237, China; Southern Marine Science and Engineer Guangdong Laboratory, Guangzhou, China; Key Laboratory of Tropical Aquatic Germplasm of Hainan Province, Sanya Oceanographic Institution, Ocean University of China, Sanya 572000, China

**Keywords:** target enrichment genotyping, probe design, DNA sequence, transformer model

## Abstract

Target enrichment sequencing techniques are gaining widespread use in the field of genomics, prized for their economic efficiency and swift processing times. However, their success depends on the performance of probes and the evenness of sequencing depth among each probe. To accurately predict probe coverage depth, a model called Deqformer is proposed in this study. Deqformer utilizes the oligonucleotides sequence of each probe, drawing inspiration from Watson–Crick base pairing and incorporating two BERT encoders to capture the underlying information from the forward and reverse probe strands, respectively. The encoded data are combined with a feed-forward network to make precise predictions of sequencing depth. The performance of Deqformer is evaluated on four different datasets: SNP panel with 38 200 probes, lncRNA panel with 2000 probes, synthetic panel with 5899 probes and HD-Marker panel for Yesso scallop with 11 000 probes. The SNP and synthetic panels achieve impressive factor 3 of accuracy (F3acc) of 96.24% and 99.66% in 5-fold cross-validation. F3acc rates of over 87.33% and 72.56% are obtained when training on the SNP panel and evaluating performance on the lncRNA and HD-Marker datasets, respectively. Our analysis reveals that Deqformer effectively captures hybridization patterns, making it robust for accurate predictions in various scenarios. Deqformer leads to a novel perspective for probe design pipeline, aiming to enhance efficiency and effectiveness in probe design tasks.

## INTRODUCTION

The advancement of next-generation sequencing (NGS) technologies has revolutionized our ability to obtain comprehensive genomic information from DNA and RNA samples. However, the high cost and time associated with conducting large-scale genomic deep sequencing studies present significant challenges. To address these challenges, target enrichment strategies have been developed, allowing researchers to focus on specific regions of interest in the genome. This approach makes deep sequencing more affordable and yields a wealth of valuable genomic data [1, 2]. Although these strategies rely on hybridization techniques to capture the regions of interest, the kinetics and thermodynamics of hybridization can lead to uneven sequencing depth across locus-specific probes [3–6], potentially resulting in increased costs due to non-uniform coverage and the necessity to enhance sequencing depth [7]. Therefore, there is a need for a computer-based approach to predict the base depth of probe sequences. Such an approach would be invaluable in aiding researchers to design probes with comparable thermodynamic characteristics, thereby achieving greater uniformity [8].

Given the substantial size of next-generation sequencing (NGS) datasets, machine learning techniques are well suited for addressing this challenge [9]. Zhang *et al*. [8] proposed a deep learning model based on a gated recurrent unit (GRU-based DLM) architecture, which utilizes DNA strand information to predict sequencing depth. The results demonstrate that the model can accurately predict sequencing depth at the same reaction condition [10]. However, the GRU-based DLM approach encounters practical limitations. It depends on global and local thermodynamic features of probes that are predicted by third-party software, in addition to the probe sequences [11]. Moreover, GRU-based DLM primarily focuses on optimizing probe selection for a specific NGS library, making its prediction results susceptible to variations resulting from different library constructions.

To overcome these limitations, we propose a novel deep learning model named Deqformer. Our model incorporates double-stranded DNA molecules into two BERT structures [12], enabling it to capture unique strand characteristics. Deqformer offers the following key advantages: (i) our model is designed to solely take oligonucleotide probes and their reverse complement sequences as input, simplifying its application and usability. (ii) The BERT structure serves as the core component of our model, allowing for the utilization of transfer learning techniques to accelerate training time. (iii) Deqformer includes an integrated gradient interpretation feature that helps infer the underlying reasons behind hybridization response predictions. This enhances the interpretability of the model, enabling a deeper understanding of the kinetics and thermodynamics involved in hybridization processes. By leveraging the high accuracy of our model, researchers can embrace new possibilities and avenues in the field of probe design. This advancement holds promising potential for advancing genomic research and enabling more accurate and efficient sequencing studies.

## MATERIALS AND METHODS

### Benchmark datasets

To evaluate the performance of Deqformer and other models, we utilized four datasets including panels of single-nucleotide polymorphisms (SNP panel), long non-coding RNA (lncRNA panel) for human, HD-Marker panel for Yesso scallops (*Mizuhopecten yessoensis*) and artificially designed synthetic panel intended for information storage (Table 1). All data were taken from previously published articles [8, 13].

**Table 1:** The probe datasets used in this study

Datasets	Number of probes	Application
HD-Marker panel	11 000	Genotyping
SNP panel	38 200	Genotyping
LncRNA panel	2000	Genotyping
Synthetic panel	5899	Information storage

As the design pipeline of probes for genomic variations profiling are quite different from artificially designed synthetic probes. We trained the models with 42 000 SNP probes and 7373 synthetic probes independently and predicted sequencing depths in cross validation for each panel individually. All the models were trained with the same splits of data. The lncRNA and HD-Marker panels served as an independent test dataset for the SNP panel, as these panels share similar reaction conditions.

### Data processing

HD-Maker probe sequences were converted to standard FASTA format and aligned to the *M. yessoensis* genomes using BWA [14]. Sequencing depth was determined using SAMtools [15]. The actual observed sequencing depths underwent a log_10_ transformation and normalization to a standard normal distribution, aimed at minimizing bias in the raw data sets. To evaluate our model, we also predicted the sequencing depths using the recently published GRU-based DLM, traditional machine learning models including XGBoost and random forest, as well as a linear regression model that correlated standard free energy and hybridization reaction temperature with sequencing depth.

### Network architecture

**Figure 1:**
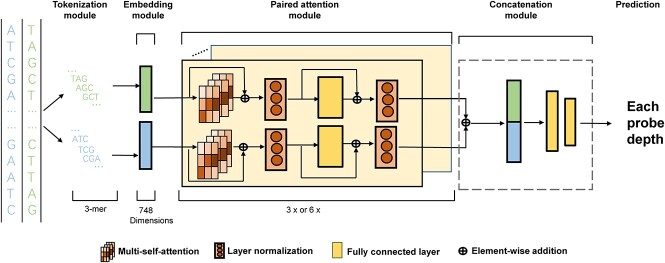
Overview of Deqformer. Deqformer consists of a tokenizer module, embedding module, paired attention module, concatenation module and predict results. Features from both the forward and reverse probe strands were extracted and concatenated to generate each probe depth.

Figure 1 shows the model structure of the Deqformer model for predicting each probe depth. We used PyTorch [16] for building the framework and implemented transformer by using the Hugging Face [17] package. The Deqformer model took oligonucleotide probes and their reverse complement sequences as input; high-dimensional information was extracted, respectively, and then combined by concatenation. Each substructure consisted of the following key modules:

(i) Tokenization module: Given the oligonucleotide sequence as input, the tokenization layer divides it into several smaller tokens. The tokenization module contains two steps; in the first step, *k*-mers are employed to produce small token. For a probe sequence of length *L*. *K*-mers are extracted use a sliding window with stride one across *s*, $\mathrm{s}\in{\Sigma}^{\mathrm{l}}$. A vector of overlapping tokens with length $L-k+1$ are produced:
  $$ K:{\varSigma}^l\to{\left({\varSigma}^k\right)}^{l-k+1},\Sigma =\left\{A,G,C,T\right\} $$
  For probe sequence $\mathrm{s}={\mathrm{s}}_1{s}_2\dots{s}_n\in{\Sigma}^l$, the result vector of tokens is $\left[{\mathrm{s}}_1\dots{\mathrm{s}}_{\mathrm{k}},{\mathrm{s}}_2\dots{\mathrm{s}}_{\mathrm{k}+1},\dots{\mathrm{s}}_{\mathrm{n}-\mathrm{k}+1}\dots{\mathrm{s}}_{\mathrm{n}}\right]$.
(ii) Embedding module: The outputs from the tokenization process are fed into an embedding module, which encompasses two types of embedding representations: token embedding and positional embedding. The token embedding maps each *k*-mer token into a 256-dimensional numerical vector. We implemented a lookup table that contained all the mappings from each token to its corresponding vector. The following position embedding function is used to represent the order of tokens:
  $$ PE\left( pos,2i\right)=\mathit{\sin}\left( pos/{1000}^{\frac{2i}{d_{model}}}\right) $$
   
  $$ PE\left( pos,2i+1\right)=\mathit{\cos}\left( pos/{1000}^{\frac{2i}{d_{model}}}\right) $$
  where *pos* means token position in DNA sequence and *i* means the dimension of the token. Token embedding and positional embedding are added to a sum embedding vector, which includes both token content information and token context information.
(iii) Paired attention module: The paired attention module (PAM) encompasses two self-attention layers [12, 18] (see Supplementary Note 1 for more details), which are specifically designed for the target probe and its reverse complement sequence, respectively. Each self-attention layer is constructed with *N* identical transformer layers, where each transformer block incorporates multi-head self-attention and a position-wise fully connected layer. Each component within the module employs a BERT model that has been pre-trained on a natural language dataset [19]. For the practical deployment, the components are implemented by invoking the DistilBertPreTrainedModel classes from the Hugging Face library.
(iv) Concatenation module: The module concatenates two branch self-attention vector into one. This design is inspired by the observation that the hybridization process is equally likely to initiate at either end, implying that a probe sequence and its reverse complement should acquire the same sequencing depth. Following concatenation layers, two feed-forward network (FFN) layers are employed to predict the sequencing depth for each probe.

### Network loss function

Mean square error (MSE) is employed as loss function, and the formula is


$$ {L}_{MSE}=\mid D\left(x;\theta \right)-{d}_{obs}{\left.\kern0em \right|}^2 $$

where $D$ means the Deqformer model, $x$ means input DNA sequence, $\theta$ means the parameters of the Deqformer model, those parameters are optimized during training stage by gradient descent algorithm and ${d}_{obs}$ is the corresponding depth of input sequence.

### Model setting and evaluation of the performance

The prediction accuracy of the model is evaluated by metrics including root mean square error (RMSE), Pearson’s correlation coefficient (*r*), Factor 2 of accuracy (F2acc) and Factor 3 of accuracy (F3acc). The metrics are calculated according to the following equations:


$$ \mathrm{RMSE}=\sqrt{{\left(\frac{1}{\mathrm{N}}{\Sigma}_{\mathrm{j}}\left({\log}_{10}\left({d}_{obs}(j)\right)-{\log}_{10}\left({d}_{pred}(j)\right)\right)\right)}^2} $$

$$ {\displaystyle \begin{array}{c}r\left(\mathrm{X},\mathrm{Y}\right)=\frac{\Sigma_{\mathrm{i}=1}^{\mathrm{n}}\left({\mathrm{x}}^{\mathrm{i}}-\hat{x}\right)\left({\mathrm{y}}^{\mathrm{i}}-\hat{y}\right)}{\sqrt{\Sigma_{i=1}^n\left({x}^i-\hat{x}\right)}\sqrt{\Sigma_{i=1}^n\left({y}^i-\hat{y}\right)}}\end{array}} $$

$$ {\displaystyle \begin{array}{c}\mathrm{F}2\mathrm{acc}=\frac{1}{\mathrm{N}}{\Sigma}_{\mathrm{j}}I\left[\left|{\log}_{10}\left({d}_{obs}(j)\right)-{\log}_{10}\left({d}_{pred}(j)\right)\right|<2\sqrt{2}\right]\end{array}} $$

$$ {\displaystyle \begin{array}{c}\mathrm{F}3\mathrm{acc}=\frac{1}{\mathrm{N}}{\Sigma}_{\mathrm{j}}I\left[\left|{\log}_{10}\left({d}_{obs}(j)\right)-{\log}_{10}\left({d}_{pred}(j)\right)\right|<3\sqrt{2}\right]\end{array}} $$

where *N* denotes the number of datasets and ${\mathrm{d}}_{\mathrm{obs}}$ and ${\mathrm{d}}_{\mathrm{pred}}$denote the observed and predicted sequencing depth of a probe sequence, respectively. RMSE reflects the magnitude of errors in probe depth, while Pearson’s *r* indicates the linear relationship between the predicted depth of probes and their actual depths. In contrast, F3acc and F2acc evaluate the proportion of accurate predictions.

The model training and evaluation are implemented in a personal computer with CPU of i7-9700 3.00 GHz, memory (RAM) of 64 GB and GeForce RTX 2080Ti.

### Model interpretation with integrated gradients and attention scores

We computed attention weights to reveal the internal activity of a model. Specifically, for each attention head within a given layer, the model generated a set of attention weight values denoted as $\mathrm{\alpha}$, where ${\alpha}_{ij}$ represents the attention from 3-mer *i* to 3-mer *j*. We then combined the set of attention weight values to produce the current attention scores of each attention head in the given layer over the dataset *X*.


$$ A={\sum}_{x\in X}{\sum}_{i=1}^{\left|x\right|}{\sum}_{j=1}^{\left|x\right|}{\alpha}_{ij} $$

Next, we plotted a heatmap by aggregating the attention weight values for all layers. The *x*-axis of the heatmap represents the attention head, and the *y*-axis denotes the encoding layer. Larger values in the heatmap indicate that the model has assigned greater attention to the corresponding positions.

The integrated gradients [20] (IGs, see Supplementary Note 2 for more details) are calculated to elucidate the learned knowledge of the model based on the provided dataset. This method combines the implementation invariance property of gradients [21], with the sensitivity of techniques to calculate the relevance score with respect to the input. Specifically, the IG value of the *i*-th 3-mer of the input sequence *x* represents the importance of that 3-mer in the overall prediction made by the model. This allows for a fine-grained interpretation of the model’s decision-making process and can help to identify which regions of the input sequence are important.

## RESULTS AND DISCUSSION

### Innovations in the framework design of Deqformer

The study introduces Deqformer, an architecture designed to model the contextualized structural properties of oligonucleotide probes and predict their sequencing depth. The architecture of Deqformer contains five main modules: tokenization, embedding, paired attention, concatenation and prediction (Figure 1).

Unlike the recently developed GRU-based DLM model, which relies on pre-selected global and local thermodynamic features, Deqformer adopts a dual BERT encoder architecture, obviating the need for manual feature extraction. The BERT-based architecture, previously successful in tasks such as speech recognition and natural language processing, allows for better capturing of long-range interactions within DNA molecules [22, 23]. This approach eliminates bias in feature extraction and curation and enables the model to be more generalizable to oligonucleotide probes of varying lengths. One notable advantage of Deqformer is its suitability for modeling asymmetric strand problems, such as bidirectional probe hybridization. This is achieved by leveraging the dual BERT encoders. In comparison to other BERT-based models in the biological field, Deqformer specifically enables strand-specific kinetic modeling. By accounting for the directionality of probe strands, our architecture enhances the accuracy and precision of predictions in scenarios involving asymmetric strand interactions.

**Figure 2:**
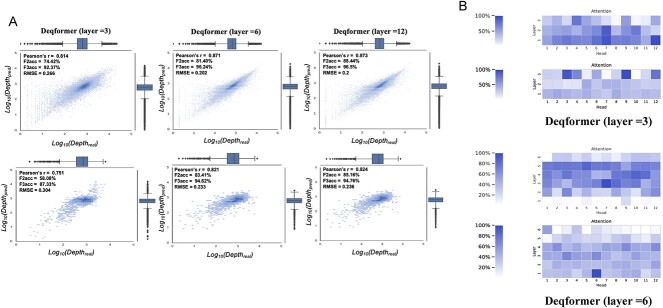
(**A**) Performance of Deqformer with different number of layers. (**B**) The two encoders of BERT attention weights of the forward and reverse strands.

To determine the optimal number of layers for the neural network structure, a grid search was performed. The results of grid search proved that using six layers can achieve smooth performance (Supplementary Figure S1 available online at http://bib.oxfordjournals.org/). Cross-validation on the SNP panel dataset demonstrated that the encoder with six layers performs comparably to the encoder with 12 layers (Figure 2A). Additionally, the result revealed that the encoder with three layers also achieved good predictive accuracy in terms of Pearson’s *r* and RMSE. This lightweight network configuration can be particularly useful in scenarios where computational resources are limited or speed is of utmost importance.

To evaluate the efficacy of the dual BERT structure, we calculated the scores of the attention weights (Figure 2B). Notably, we observed distinct attention weights for the two encoders, implying that each encoder can extract different features. This underscores the significance of the Deqformer structure, regardless of whether it consists of three or six layers. The dual BERT encoders architecture can effectively capture both global and local information from the DNA sequences, which is especially well suited for asymmetric strand problems, such as bidirectional probe hybridization. Deqformer offers users the flexibility of choosing lightweight computation options and enabling users to select the optimal configuration that caters to their unique requirements.

### Performance of Deqformer on predicting probe depth

**Figure 3:**
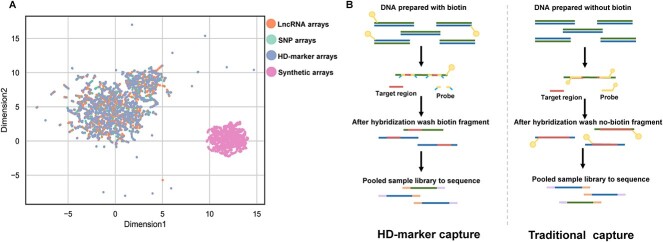
(**A**) Uniform manifold approximation and projection (UMAP) of four datasets. (**B**) Libraries preparation of HD-Marker (left) and targeted DNA sequencing (right).

For assessing the performance of Deqformer in predicting NGS depth, we compared its performance with the XGBoost [24], random forest [25], linear regression model and the GRU-based DLM. The SNP and synthetic panels were used for 5-fold cross validation. To establish a more robust baseline, we retrained the GRU-based DLM along with other models using the same data splits. This retraining process resulted in a slight decrease in the performance of the GRU-based DLM on the SNP panel. The F2acc and F3acc metrics dropped to 76.25% and 90.12%, respectively, compared to the previously reported values of 79.01% for F2acc and 92.69% for F3acc [8]. However, the performance on the synthetic panel remained unchanged. As there are variations in experimental variables associated with probe synthesis, target genome preparation and library construction among the four panels, we employed uniform manifold approximation and projection (UMAP) to assess the overall data structure. The UMAP visualization demonstrates clear stratification between the synthetic and the other three panels (Figure 3A). Therefore, we trained the model with the SNP and synthetic panel separately.

**Figure 4:**
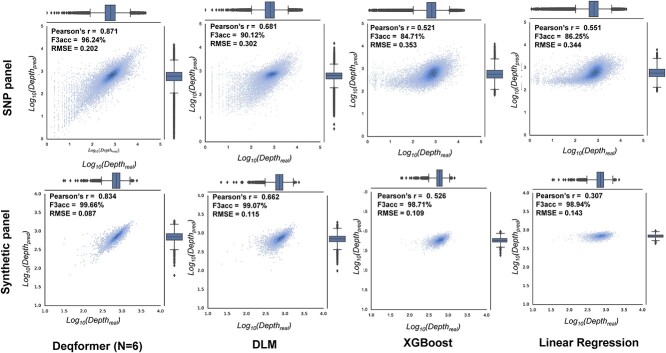
Cross-validation on training datasets. Predicted versus observed sequencing depth for the two panels of the three models.

Each probe of the SNP panel consists of an 80 bp target-specific sequence and two 30 bp adapters at each end. After quality control, a total of 38 040 probes from the SNP panel were kept for subsequent analysis. Deqformer demonstrated notable performance in sequence depth prediction, achieving a Pearson’s *r* of 0.871, an RMSE of 0.202, an F2acc of 85.16% and an F3acc of 96.24% (Figure 4, Table 2). For comparison, the GRU-based DLM, linear regression and XGBoost models yielded Pearson’s *r* values of 0.681, 0.551 and 0.521, respectively, along with RMSEs of 0.302, 0.344 and 0.353. These models also exhibited higher bias compared with Deqformer (Supplementary Figure S2 available online at http://bib.oxfordjournals.org/ and Table 2). We additionally measured the uncertainty in cross-validation with the SNP dataset. The standard deviation of RMSE indicated that our model exhibited comparable generality performance to other models, without any discernible negative impact (Supplementary Table S1 available online at http://bib.oxfordjournals.org/).

**Table 2:** Performance of different models on the SNP and synthetic probe panels

Datasets	Model	RMSE	F2acc(%)	F3acc(%)	Pearson’s *r*
SNP panel	Random forest	0.406	63.82	81.10	0.263
	Linear regression	0.344	69.97	86.25	0.551
	XGBoost	0.353	67.97	84.71	0.521
	GRU-based DLM	0.302	76.25	90.12	0.681
	Deqformer	0.202	85.16	96.24	0.871
Synthetic panel	Random forest	0.399	56.83	76.08	0.246
	Linear regression	0.143	95.49	98.94	0.308
	XGBoost	0.109	97.11	98.71	0.526
	GRU-based DLM	0.115	97.56	99.07	0.662
	Deqformer	0.087	98.97	99.66	0.834

Regarding the synthesis dataset, where all probes share a sequence length of 110 bp, our Deqformer model also outperformed other models (Table 2). Deqformer attains a Pearson’s *r* of 0.834, an RMSE of 0.087, an F2acc of 98.97% and an F3acc of 99.66% in comparison to the observed depth (Figure 4, Table 2). By contrast, the GRU-based DLM model achieves a Pearson’s *r* of 0.662 and an RMSE of 0.115, while the linear regression model exhibits a Pearson’s *r* of 0.307 and an RMSE of 0.143. Furthermore, the XGBoost model demonstrates a Pearson’s *r* of 0.526 and an RMSE of 0.109 (Figure 4, Table 2, Supplementary Figure S2 available online at http://bib.oxfordjournals.org/).

### Generativity of Deqformer

**Figure 5:**
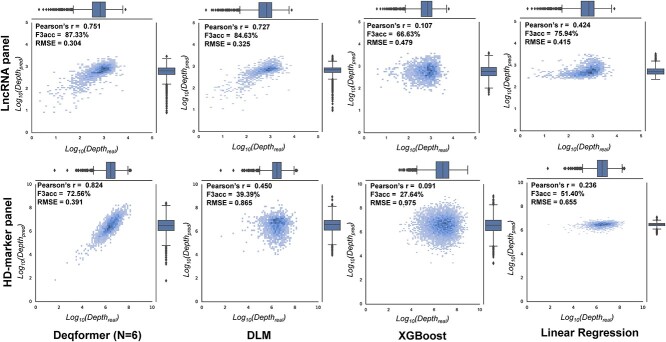
Evaluation by independent datasets. LncRNA and HD-Marker panel were tested as two independent datasets. Predicted versus observed sequencing depth for the two probe panels of the three models.

To assess the performance of Deqformer on independent panels, we selected lncRNA and HD-Marker panels as test datasets. Our results indicated that after training with 100 epochs, Deqformer showed good generalizability on the lncRNA probes. The predicted results achieved a Pearson’s *r* of 0.751, RMSE of 0.304, F2acc of 70.80% and F3acc of 87.33% (Figure 5, Supplementary Table S2 available online at http://bib.oxfordjournals.org/). In comparison, the GRU-based DLM, XGBoost and linear regression models displayed lower correlation coefficients and higher RMSEs (Figure 5, Supplementary Table S2 available online at http://bib.oxfordjournals.org/). We also evaluated the robustness of Deqformer using the HD-Marker dataset. Compared with the traditional target enrichment genotyping method, the HD-Marker panel has different experimental processes of probe synthesis, target genome preparation and library construction. It utilizes two flanking probes for target binding (Figure 3B). Our results demonstrated that Deqformer was robust in predicting the sequencing depth of HD-Marker panel, with a Pearson’s *r* of 0.824, RMSE of 0.391, F2acc of 66.63% and F3acc of 72.56% (Figure 5, Supplementary Table S2 available online at http://bib.oxfordjournals.org/). Although there is a slight decrease in performance compared to the lncRNA dataset, Deqformer still outperforms the GRU-based DLM, XGBoost and linear regression models. Deqformer's consistent outperformance over the other three models highlights its cutting-edge design and effectiveness. Linear regression models have been prone to underfitting in complex genomic landscapes due to their simplicity [26]. While XGBoost, as a gradient boosting framework, excels in handling a variety of data types and complex structures, it can sometimes be overfit on noisy datasets. They might not always encapsulate the intricate relationships present in genomic data. Deqformer's integration of presumably deeper or more representative features offers a leap forward.

### Performance on identifying high depth probe

**Figure 6:**
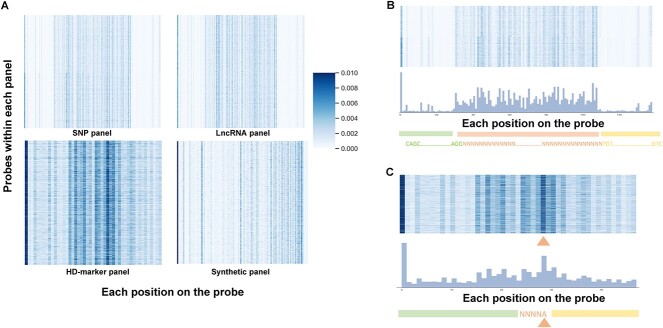
(**A**) The input gradients distribution of Deqformer on four panels. (**B**) The IG values of SNP panel. (**C**) The IG values of HD-Marker panel.

To analyze the contribution of different probe sets in the model’s prediction, we utilized Captum [27] to compute the input gradients of the four panels. The results illustrated that nucleotides located in the middle of the probe had a noticeably higher weight compared to the others on the probe (Figure 6A). Except the synthetic panel, the probes from the other three datasets exhibited a similar pattern, which is in concordance with the UMAP analysis. Furthermore, we analyzed the IG values specifically for the SNP probe set and find that the middle 80 bp position showed relatively higher weights, while the extension arms at both ends displayed lower IG values (Figure 6B). Similarly, for the HD-Marker probe set, the probe weight plot shows that heavier weights correspond well to the probe capture region, with the highest weight at the target SNPs site (Figure 6C). These results indicate that our model effectively captures the unique characteristics of different probe sets and learns distinct weight information for their prediction. The observation that nucleotides at the end of the probe bear a higher weight is compelling, hinting that these regions may play a crucial role in determining the probe’s depth. This could be due to their direct interaction with their target or potential susceptibility to experimental nuances. Concurrently, the unique weight patterns observe for the SNP probe set and the HD-Marker probe set illuminate the model’s adaptability and precision. Such distinct patterns, in a biological and clinical frame, could be linked to pivotal functional roles, ranging from the efficacy of target capture to the specificity of interactions [28]. For instance, the pronounced weight at the target SNP site within the HD-Marker set possibly emphasizes the significance of these genetic markers in genotyping processes. Delving into literature studies, especially those centers on probe–target dynamics, could shed more light on these intriguing positional nuances and the broader implications they carry for genomics.

### Ablation study on the components of Deqformer

We conducted an ablation study to assess the efficacy of the key components of Deqformer: *k*-mer tokenization and the use of both oligonucleotide probes and their reverse complement sequences inputs. To establish a robust baseline, we implemented BERT with BPE tokenization, while deliberately excluding the use of *k*-mer tokenization and the PAM to better discern the effects of these components. Substituting BPE with *k*-mer tokenization led to a significant improvement, as evidenced by a decrease in RMSE values from 0.351 to 0.318, an increase in F2acc from 70.80% to 74.89%, a rise in F3acc from 85.38% to 88.44% and an enhancement in Pearson’s *r* from 0.520 to 0.639, respectively (Table 3, Supplementary Figure S3 available online at http://bib.oxfordjournals.org/). This change clearly demonstrates the positive impact of *k*-mer tokenization on predictive accuracy.

**Table 3:** Ablation study of Deqformer model

*K*-mer tokenization	PAM	RMSE	F2acc (%)	F3acc (%)	Pearson’s *r*
−	−	0.351	70.80	85.38	0.520
+	−	0.318	74.89	88.44	0.639
+	+	0.202	85.16	96.24	0.871

We further integrated PAM into the model architecture, which led to obvious enhancements: RMSE, F2acc, F3acc and the Pearson’s *r* reach values of 0.202, 85.16%, 96.24% and 0.871, respectively (Table 3, Supplementary Figure S3 available online at http://bib.oxfordjournals.org/). This nuanced evaluation not only underscores the efficacy of Deqformer's individual components but also provided a comprehensive understanding of their synergistic effect on predictive performance.

### Revelations of thermodynamic properties of probe hybridization

In the field of probe design research, kinetics and thermodynamics are recognized as crucial factors in the hybridization process. To assess the neural network’s learning of these factors, we used Primer3 [29] to extract thermodynamics metrics, including the stability of secondary structure and Gibbs free energy of the probes. We subsequently computed the Kendall-τ coefficients to assess the correlation between IG values and thermodynamic metrics. The mean Kendall-τ coefficients for IG in relation to free energy, and IG in relation to secondary structure, were found to be 0.210 and 0.276, respectively (Supplementary Table S3 and Supplementary Figure S4 available online at http://bib.oxfordjournals.org/). These results indicate a consistent and positive correlation, wherein the rank order of nucleotide compositions based on their importance moderately aligns with variations in thermodynamic properties. However, the correlations are not sufficiently strong to suggest high predictability, implying that our model might have captured more nuanced information within the probe sequences [30, 31].

Probes from the four panels were identified as extremely high and low depths drawn at a rate of 5% using a Gaussian distribution. The frequency of 3-mer combinations with extremely high or low depths were compared with the total datasets (Supplementary Figure S5 available online at http://bib.oxfordjournals.org/), we observed that there were no commonalities for the probes with extremely low depth whereas the extremely high-depth combinations showed a higher content of CT (~35%). These findings suggest that non-polar amino acid combinations may be more susceptible to sequencing errors, supporting a previous study that indicated interference between non-polarity and Watson–Crick base pairing [32].

The design of oligonucleotide probes is often based on GC content and Tm values which the basic parameter that associated with the hybridization kinetics and thermodynamics [33]. It is worth noting that previous probe design software, like BaitFisher [34] and CATCH [35], focuses on accurately recognizing multi-species sites during probe design. While HUBDesign [36] improves sequencing uniformity, it still does not completely address the issue of uneven sequencing, whereas the Deqformer model provides an alternative approach that accurately predicts probe depth by using probe sequences. By integrating the Deqformer model into the probe design workflow, probes with extremely high or low predicted sequencing depth are easily filtered. Since Deqformer only requires the probe sequence as input, it can be easily integrated with other software tools and assist in their probe design processes (Supplementary Figure S6 available online at http://bib.oxfordjournals.org/). Given the insights into probe behavior, subsequent studies could focus on experimentally validating these findings.

## CONCLUSION

In this study, we introduce a novel end-to-end model that predicts read depth for probe sequences. Our method demonstrates its good performance across various datasets, including SNP, lncRNA, syntenic and HD-Marker panels according to multi-metrics, confirming that our proposed model achieves state-of-the-art results. One notable advantage of our model is its ability to identify probes with extremely high or low sequencing depth. This feature empowers probe designers to efficiently select probes that meet their specific requirements. Furthermore, the open-source nature of our model enables easy integration with existing probe design software. This accessibility facilitates its adoption by the scientific community, making it a valuable tool for genetic studies. Deqformer effectively captures hybridization patterns, making it robust for accurate predictions in various scenarios. By embracing this new perspective, we anticipate significant improvements in both the accuracy and efficiency of genomic analysis as more researchers adopt this method and develop more efficient probes. With its focus on sequencing depth, it offers a fresh approach that holds potential for advancing genomic analysis and facilitating a deeper understanding of complex biological systems.

**Key Points**

- In this study, an oligonucleotide probes design framework for targeted high-throughput DNA sequencing named Deqformer is developed, which can accurately predict the sequencing depth of each probe using a transformer-based deep learning neural network architecture.
- Deqformer is designed to solely take oligonucleotide probe sequences as input, which greatly simplify its application compared with previously publicized linear regression and recurrent neural network models.
- Deqformer exhibits promising performance, achieving factor 3 of accuracy (F3acc) of 96.24% and 99.66% in cross-validations of the SNP and synthetic panels, respectively. In independent tests of the lncRNA and HD-Marker panels, its F3acc reached 87.33% and 72.56%, respectively.
- The Deqformer model incorporates a gradient interpretation feature that aids in inferring the underlying factors contributing to the predictions related to hybridization response. This feature enhances the model’s interpretability, promoting a deeper understanding of the kinetics and thermodynamics associated of probe hybridization.

## Supplementary Material

Supplementary_files_bbae007

## Data Availability

Deqformer is available on GitHub at https://github.com/Deqformer/Deqformer-code. The HD-Marker datasets can be retrieved from the NCBI Sequence Read Archive (SRA) at https://www.ncbi.nlm.nih.gov/sra under the accession numbers SRP115866, SRP115869-SRP115871 and SRP115873.

## Author Biographies

**Yantong Cai** is a PhD student in the MOE Key Laboratory of Marine Genetics and Breeding, College of Marine Life Sciences, Ocean University of China. Her research focuses on the application of deep learning in bioinformatics.

**Jia Lv** is an associate professor in the MOE Key Laboratory of Marine Genetics and Breeding, College of Marine Life Sciences, Ocean University of China. Her research centers on development of genomic techniques.

**Rui Li** is a PhD student in the MOE Key Laboratory of Marine Genetics and Breeding, College of Marine Life Sciences, Ocean University of China. Her research mainly focuses on genomics.

**Xiaowen Huang** is a PhD student in the MOE Key Laboratory of Marine Genetics and Breeding, College of Marine Life Sciences, Ocean University of China. Her research has centered on development of genomic techniques.

**Shi Wang** is a professor in the MOE Key Laboratory of Marine Genetics and Breeding, College of Marine Life Sciences, Ocean University of China. His research interests are centered on marine genomics and evo-devo.

**Zhenmin Bao** is a professor in the Southern Marine Science and Engineer Guangdong Laboratory. His research centers around shellfish genetics and breeding.

**Qifan Zeng** is an associate professor in the MOE Key Laboratory of Marine Genetics and Breeding, College of Marine Life Sciences, Ocean University of China. His research primarily centers around genomics and bioinformatics.
