# Supplementary material for: Deqformer: high-definition and scalable deep learning probe design method
Source: Brief Bioinform. 2024 Feb 1;25(2):bbae007. doi: 10.1093/bib/bbae007 (PMC10835675; doi:10.1093/bib/bbae007)
Supplement: Supplementary_files_bbae007 [file supplementary_files_bbae007.docx]

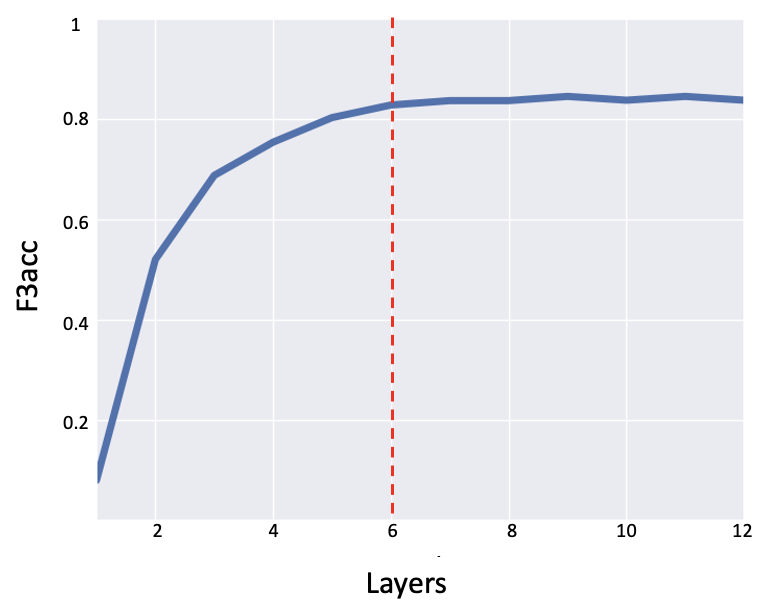
**Figure S1**. The grid search results of each layer of the Deqformer model.


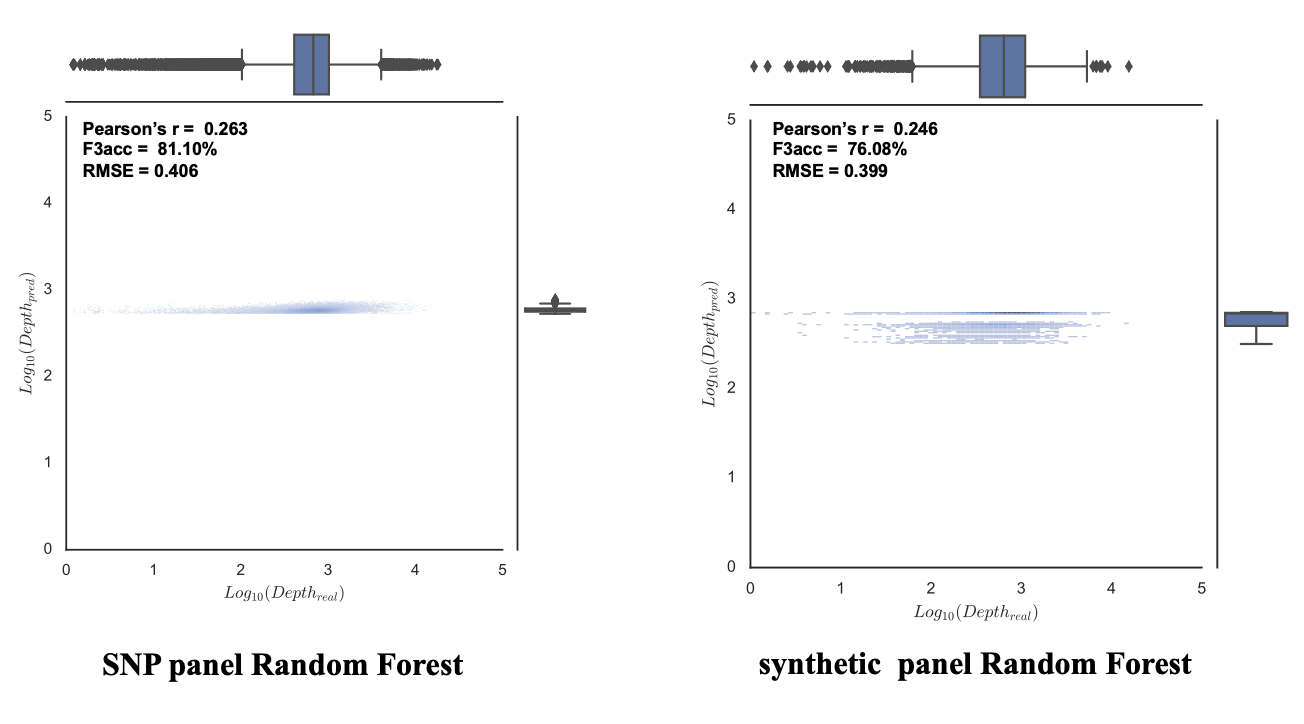


**Figure S2**. The performance of the random forest model on the SNP and synthetic probe panels.


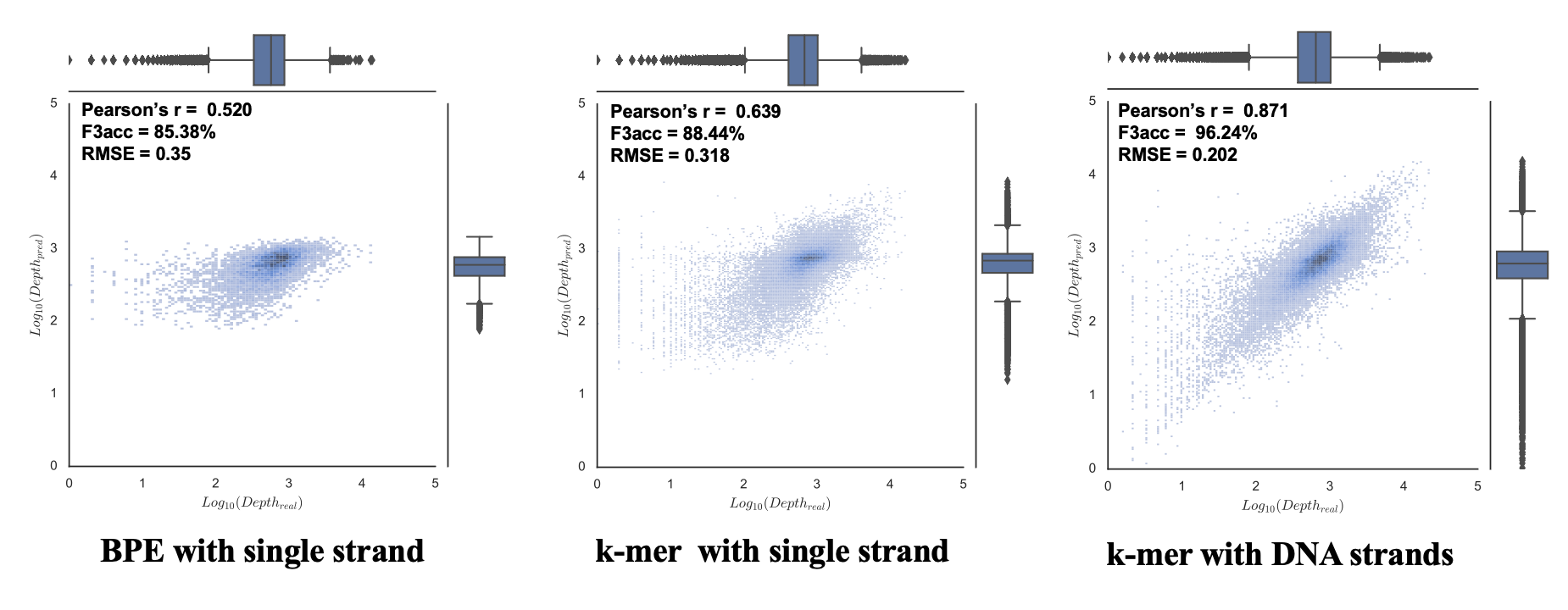


**Figure S3**. Ablation analysis of the Deqformer model.


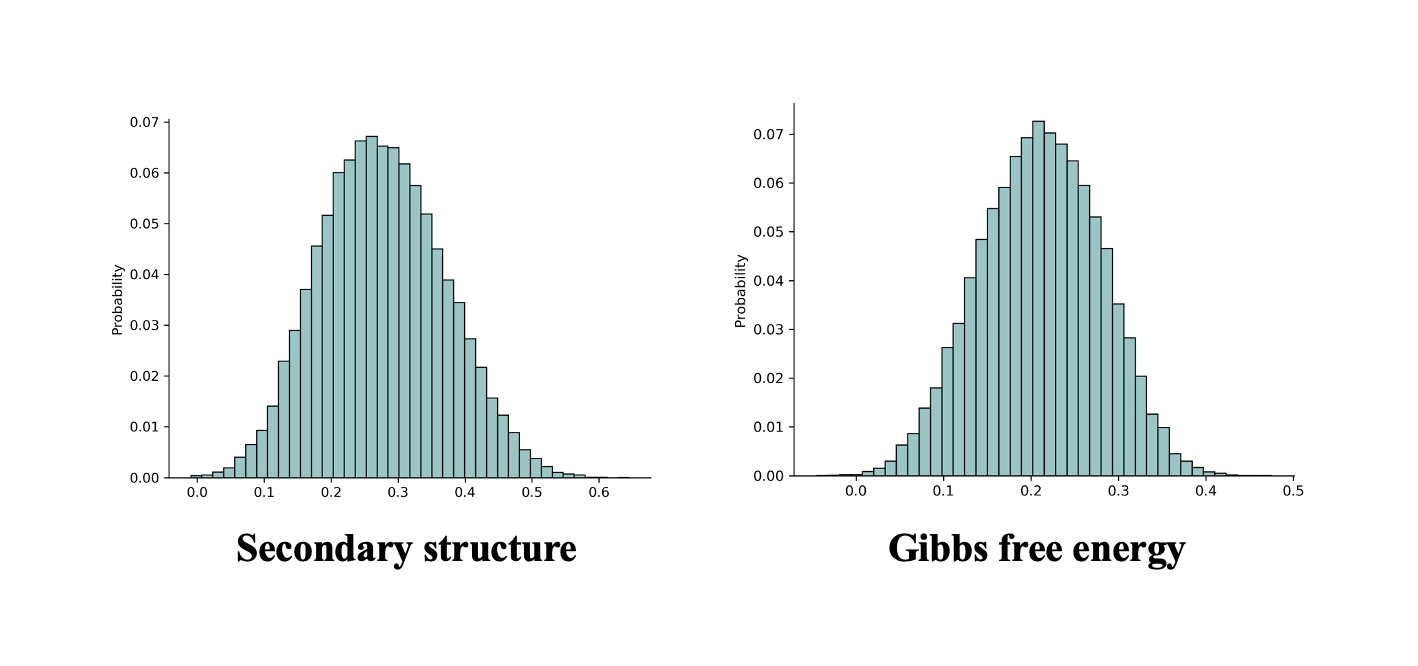


**Figure S4**. Distributions of the Kendall-τ coefficients of the stability of secondary structure and Gibbs free energy.


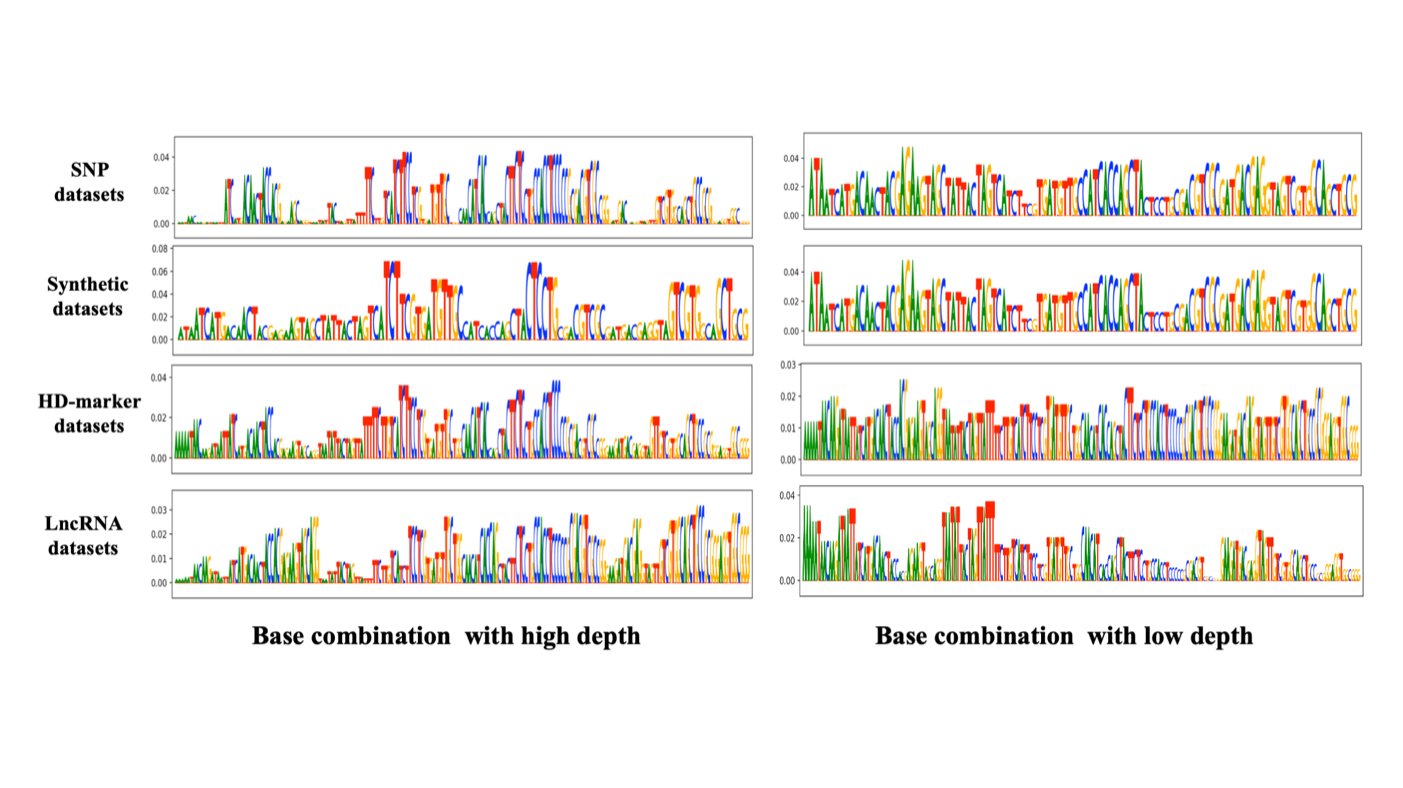
 **Figure S5.** Sequence characteristics of oligonucleotide probes with excessively high and low sequencing depths in the four datasets.


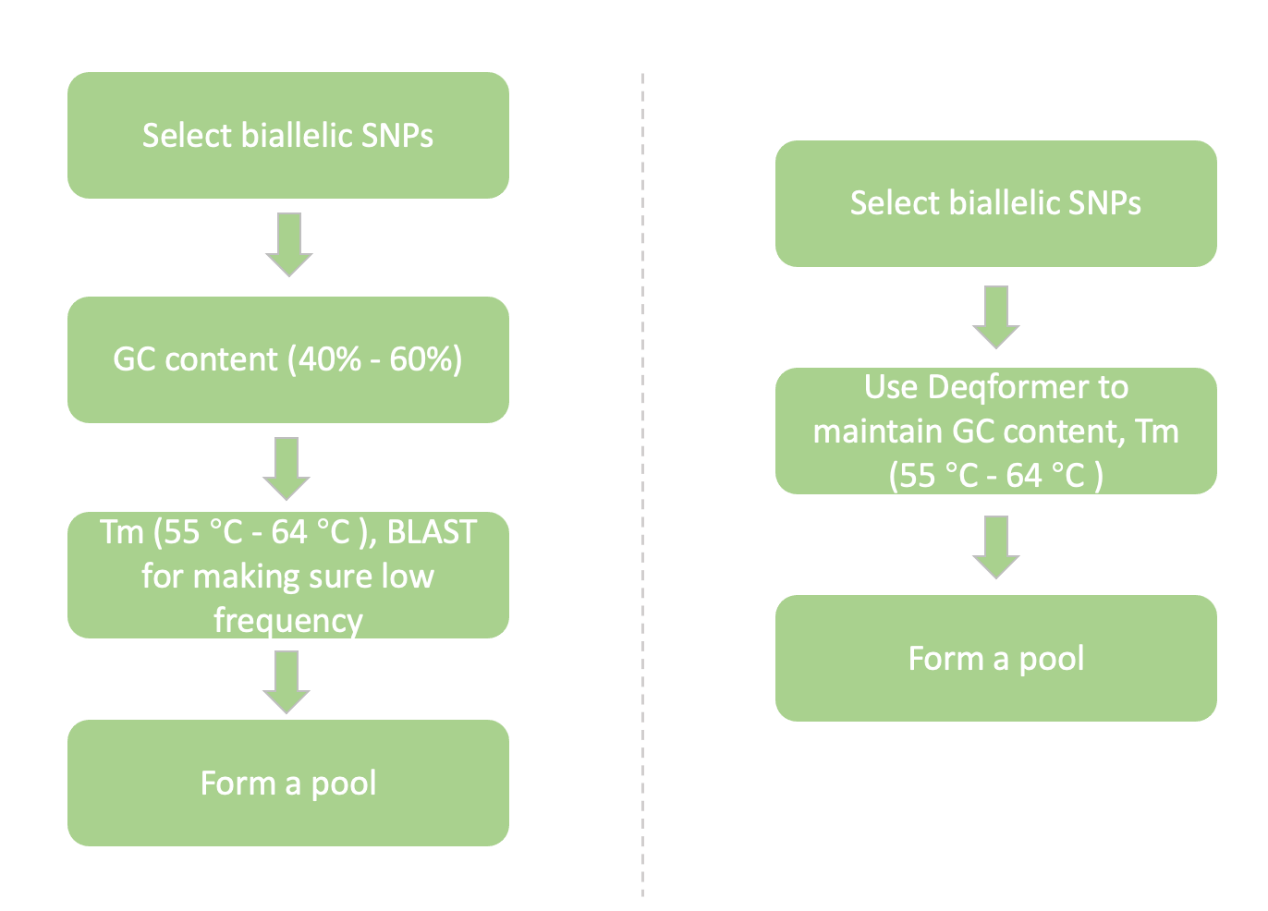


**Figure S6.** The updated probe design pipeline by integrating Deqformer.

**Table S1.** The uncertainty measures of cross-validation.

| Model | RMSE↓ |
| --- | --- |
| XGBoost | $0.357\pm0.0031$ |
| Random forest | $0.406\pm0.0007$ |
| Linear regression | $0.344\pm0.0044$ |
| GRU-based DLM | $0.302\pm0.0077$ |
| Deqformer | $0.278\pm0.0068$ |

**Table S2.** Performance of different models on the lncRNA and HD-marker probe panels.

| Datasets | Model | RMSE↓ | F2acc(%)↑ | F3acc(%)↑ | Pearson’s r↑ |
| --- | --- | --- | --- | --- | --- |
| lncRNA  panel | Linear Regression | 0.424 | 56.45 | 75.94 | 0.415 |
|  | XGBoost | 0.479 | 48.77 | 66.63 | 0.107 |
|  | GRU-based DLM | 0.325 | 66.93 | 84.63 | 0.727 |
|  | Deqformer | **0.304** | **70.80** | **87.33** | **0.751** |
| HD-marker panel | Linear Regression | 0.655 | 35.42 | 51.40 | 0.236 |
|  | XGBoost | 0.975 | 18.59 | 27.64 | 0.091 |
|  | GRU-based DLM | 0.865 | 26.32 | 39.39 | 0.450 |
|  | Deqformer | **0.391** | **66.63** | **72.56** | **0.824** |

**Table S3.** The Kendall-τ coefficients of integrated gradients (IG) scores between free energy and secondary structure.

| Kendall | Mean$\pm$Std |
| --- | --- |
| Kendall-$\tau_{free-energy}$ | 0.210$\pm$0.069 |
| Kendall-$\tau_{secondary-structure}$ | 0.276$\pm$0.092 |

**Supplementary Notes**

**1. Self-attention layer details**

Self-attention can be modeled by a function mapping input sequence $x=\left\{ x_{1},\ldots,x_{n} \right\}$ where $x\in R^{d_{x}}$ to output sequence $z=\left\{ z_{1},\ldots,z_{n} \right\}$, attention function divide input *x* into query *Q*, key *K* and value *V* vectors, these three vectors are linear transformation of input *x*, and output *z* is weighted sum of three linear transformation *Q, K* and *V*. The formula was as follows:

$$z_{i}=\sum_{j=1}^{n} a_{ij}\left( W^{V}x_{j} \right)$$

$$a_{ij}=\frac{\exp\left( e_{ij} \right)}{\sum_{k=1}^{n} \exp\left( e_{ik} \right)}$$

$$e_{ij}=\frac{\left( x_{i}W^{Q} \right)\left( x_{j}W^{K} \right)^{T}}{\sqrt{d_{k}}}$$

Multiple head attention is used in Deqformer, multiple different attention projection is concatenated into one vector. It can be formula as:

$$MultiHead\left( x \right)=Concat({Attention}_{1}(x),\ldots,{Attention}_{n}(x))$$

Where Attention is attention function, and n is the number of Attention heads.

**2. Integrated Gradients**

Specifically, assuming that the Deqformer model is denoted by F, the integrated gradients approach computes the gradients at all points along the path from the baseline input $x^{'}$ (which is the zero vector) to the input x, and then aggregates these gradients to obtain a comprehensive view of the importance of each input feature. The mathematical expression of the integrated gradients method is as follows:

$${IntegratedGrads}_{i}\left( x \right)=\left( x_{i}-x_{i}^{'} \right)\times\int_{\alpha=0}^{1} \frac{\partial F\left( x^{'} \right)+\alpha\times\left( x-x^{'} \right)}{\partial x_{i}}d\alpha$$

To generate the Integrated Gradients (IG) scores, the trained model F, the input sequence x, and the probe depth are required. The IG values are computed as a function of these inputs and have the same shape as the input sequence x.
